# C-Phycocyanin Confers Protection against Oxalate-Mediated Oxidative Stress and Mitochondrial Dysfunctions in MDCK Cells

**DOI:** 10.1371/journal.pone.0093056

**Published:** 2014-04-01

**Authors:** Shukkur M. Farooq, Nithin B. Boppana, Devarajan Asokan, Shamala D. Sekaran, Esaki M. Shankar, Chunying Li, Kaliappan Gopal, Sazaly A. Bakar, Harve S. Karthik, Abdul S. Ebrahim

**Affiliations:** 1 Department of Pharmacy Practice, Eugene Applebaum College of Pharmacy and Health Sciences, Wayne State University, Detroit, Michigan, United States of America; 2 Department of Pharmaceutical Sciences, Eugene Applebaum College of Pharmacy and Health Sciences, Wayne State University, Detroit, Michigan, United States of America; 3 Department of Medicine, University of California Los Angeles, Los Angeles, California, United States of America; 4 Department of Medical Microbiology, Faculty of Medicine, University of Malaya, Kuala Lumpur, Malaysia; 5 Tropical Infectious Diseases Research and Education Center (TIDREC), Department of Medical Microbiology, Faculty of Medicine, University of Malaya, Kuala Lumpur, Malaysia; 6 Department of Biochemistry and Molecular Biology, Wayne State University, School of Medicine, Detroit, Michigan, United States of America; 7 Department of Orthopedics, National Orthopedics Center for Excellence in Research and Learning (NOCERAL), Faculty of Medicine, University of Malaya, Kuala Lumpur, Malaysia; 8 Department of Anatomy, Yong Loo Lin School of Medicine, National University of Singapore, Singapore, Singapore; 9 Department of Internal Medicine, Wayne State University, Detroit, Michigan, United States of America; University of Pittsburgh, United States of America

## Abstract

Oxalate toxicity is mediated through generation of reactive oxygen species (ROS) via a process that is partly dependent on mitochondrial dysfunction. Here, we investigated whether C-phycocyanin (CP) could protect against oxidative stress-mediated intracellular damage triggered by oxalate in MDCK cells. DCFDA, a fluorescence-based probe and hexanoyl-lysine adduct (HEL), an oxidative stress marker were used to investigate the effect of CP on oxalate-induced ROS production and membrane lipid peroxidation (LPO). The role of CP against oxalate-induced oxidative stress was studied by the evaluation of mitochondrial membrane potential by JC1 fluorescein staining, quantification of ATP synthesis and stress-induced MAP kinases (JNK/SAPK and ERK1/2). Our results revealed that oxalate-induced cells show markedly increased ROS levels and HEL protein expression that were significantly decreased following pre-treatment with CP. Further, JC1 staining showed that CP pre-treatment conferred significant protection from mitochondrial membrane permeability and increased ATP production in CP-treated cells than oxalate-alone-treated cells. In addition, CP treated cells significantly decreased the expression of phosphorylated JNK/SAPK and ERK1/2 as compared to oxalate-alone-treated cells. We concluded that CP could be used as a potential free radical-scavenging therapeutic strategy against oxidative stress-associated diseases including urolithiasis.

## Introduction

Urolithiasis is a complex disease characterized by the formation of stones in the urinary tract [1]. Accumulating lines of evidence suggest that renal tubular cell injury and fixed crystal particles could be implicated in the pathogenesis of urolithiasis [2]–[4]. Others have postulated that excessive excretion of urinary oxalate could cause substantial damage to the renal epithelium [5]. Others have reported that oxalate generates excessive free radicals leading to renal epithelial cell injury and membrane lipid peroxidation (LPO), which in turn favors urolithogenesis [6]. Exposure to oxalate, a major component of kidney stones, elicits a cascade of responses in renal epithelial cells that often leads to cell injury or death [7], [8]. Numerous studies have suggested that oxalate toxicity is accompanied by the generation of reactive oxygen species (ROS) in renal cell cultures [9], [10]. Oxalate exposure also imposes oxidative stress on renal cells by stimulating accumulation of lipid peroxides [11] while decreasing the availability of other cellular antioxidants [12].

ROS are generated as byproducts of electron transport in mitochondria [13]–[15]. Intracellular and intra-mitochondrial antioxidants prevent cellular damage due to endogenous ROS, although conditions that increase ROS generation or diminish antioxidant availability could increase intracellular accumulation of ROS. Accumulating body of evidence suggests that mitochondria represent an important source of ROS, produced in renal cells following exposure to oxalate [16], [17]. Thus, mitochondria could be a key mediator of pathogenesis in oxalate exposure, increasing mitochondrial ROS production, which however remains to be investigated.

Several antioxidant drugs have been examined for their ability to prevent ROS-induced nephronal cell death, such as vitamin E [18], quercetin [19], lipoic acid, desferoxamine [20] and green tea [21]. The search for agents with antioxidant and nephroprotective action could therefore be of paramount importance in nephrology research. Previously, we have demonstrated that C-phycocyanin (CP), a biliprotein pigment found in the blue-green algae *Spirulina spp.* prevents the nephrotoxic effects of oxalate under *in vivo* conditions [22]–[24]. Furthermore, other reports have indicated that CP has antioxidant [25], [26], anti-inflammatory and hepatoprotective [27], free radical-scavenging [28], [29] and neuroprotective effects [30], [31]. Evidence is accumulating on the hydroxyl and peroxyl free radical-scavenging properties of CP suggesting that its therapeutic effects are largely attributed to its antioxidant potentials [32]. Here, we investigated the potential functions of CP in preventing Madin-Darby canine kidney (MDCK) cells against oxalate-induced free radical production. Our results showed that CP could significantly inhibit oxalate-induced free radical production and LPO as assessed by DCF-DA assay and HEL western blot, respectively. Furthermore, we also established that CP maintains the integrity of mitochondrial membrane potential and ATP production as evaluated by JC-1 staining and ATP bioluminescence assays, respectively. In addition, we also showed that certain oxalate-mediated stress-induced kinases, such as ERK and JNK were ameliorated by CP. Thus, CP could be a potential therapeutic strategy for oxidative stress-associated diseases such as urolithiasis, neurodegenerative disorders and aging.

## Results

### C-phycocyanin Treatment Improves Viability of MDCK Cells Exposed to Oxalate

**Figure 1:**
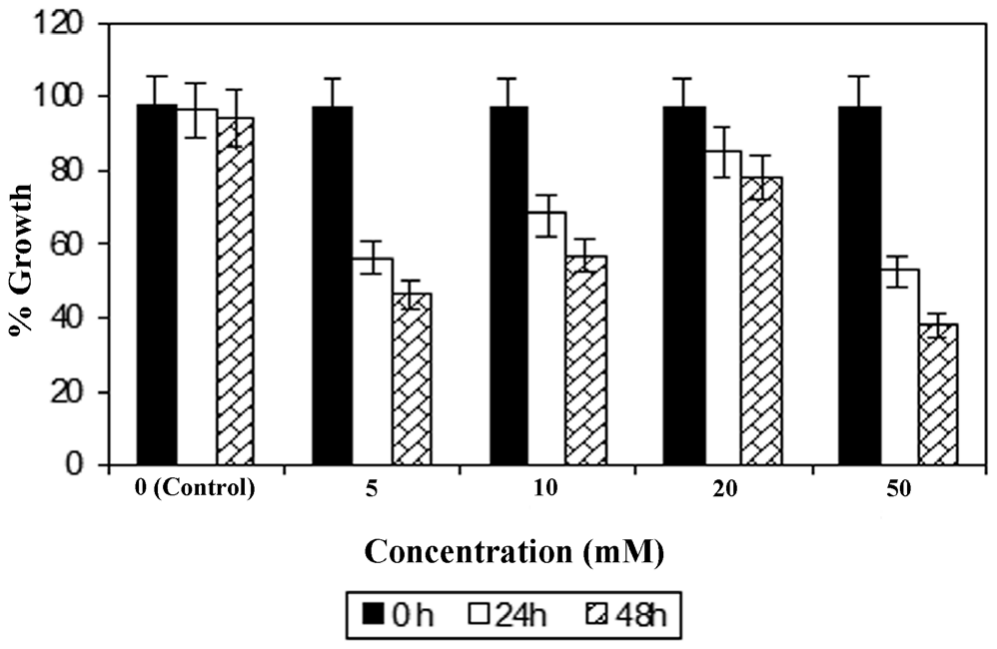
Concentrations of C-phycocyanin in the presence of oxalate in MDCK cells. Different concentrations of CP (5, 10, 20, 50 mM) in the presence of oxalate (0.1 mM) in MDCK cell line showing the effective dosage of CP at 20 mM concentration at 24 and 48 hours.

To test the effectiveness of the dosage of CP on oxalate exposure, MDCK cells were incubated with different concentrations of oxalate such as 0.02, 0.04, 0.06, 0.08 and 0.1 mM. Amongst these, we found that 0.1 mM oxalate dosage was most effective (**data not shown**). We measured cell viability using the MTT assay with this concentration of oxalate (0.1 mM). About 1 hour prior to oxalate exposure, cells were treated with four different concentrations of CP (5, 10, 20, and 50 mM) and examined at 0, 24 and 48 hours to check cell viability. As evident from **Fig. 1**, treatment with 20 mM CP significantly increased the viability of MDCK cells following 24 and 48 hours of exposure to oxalate. Further, 50 mM CP resulted in the strongest cell death both at 24 and 48 hours suggesting that high dose of CP was cytotoxic *in vitro*. Of note, we recently optimized a CP dose concentration of 100 mg/kg for oxalate-induced urolithiasis *in vivo* mouse model [22]–[24]. Another report suggested that 100 mg/kg of CP could function as the effective dose in kainic acid-induced neuronal damage in rat hippocampus [33]. Hence, extensive research may be required to determine the effective dose of CP for potential use in the treatment of human nephrolithiasis.

### Pre-Treatment of MDCK Cells Exposed to Oxalate with C-phycocyanin Attenuates Free Radical Synthesis

To validate the generation of free radicals by oxalate, we introduced a free radical sensitive compound DCF-DA (2′, 7′-dichloro-dihydro-fluorescein-diacetate) into MDCK cells. In native state, DCF-DA does not have any appreciable fluorescence of its own. However, in the presence of free radicals, the DCF-DA gets converted to a fluorogenic product, DCF (dichlorofluorescein) by oxidative cleavage exhibiting a brilliant fluorescence at 520 nm following excitation at 485 nm. The metabolic center of cellular activity being mitochondria, it would not be hard to conceptualize that the site of free radical generation is this very organelle. Therefore, the observation of increased DCF fluorescence is an evidence for heightened free radical generation within the cells.

**Figure 2:**
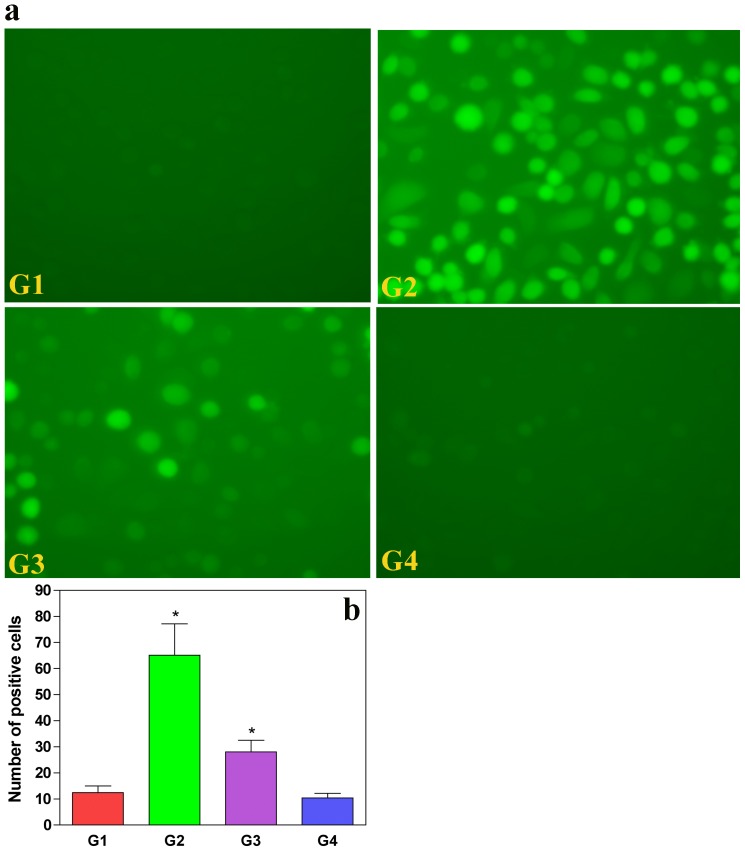
C-phycocyanin quenches oxalate-induced ROS production in MDCK cells. (**A**) MDCK cells treated with oxalate were observed for ROS generation with a non-fluorescent dye DCFDA, which converts to a fluorescent DCF compound during oxidative stress (panel G2). This is compared with panel G1 (normal conditions). Panel G3 shows reduced fluorescence due to reduced generation of ROS upon CP pre-treatment, which resembles normal cells. Panel G4 confirms that CP alone by itself is not cytotoxic. (**B**) Quantitative estimation of DCF fluorescence, oxalate-induced MDCK cells (G2) shows exaggerated fluorescence as compared to control, and reduced florescence was observed in the CP pre-treated cells (G3). Values are expressed as mean±S.D. P<0.05 were considered significant. *G2 compared with G1; G3 compared with G2. G1: Control; G2: Oxalate; G3: Oxalate+CP; G4: CP alone.

The MDCK cells treated with oxalate showed a marked increase in DCF fluorescence, thus revealing the ability of oxalate to enhance free radical generation within live cells (**Fig. 2G2**) heralding the onset of membrane damage. We observed that the non-oxalate-treated cells did not reveal any appreciable fluorescence (**Fig. 2G1**). However, pre-treatment of CP with oxalate showed a definitive significant decrease in the fluorescence of DCF (**Fig. 2G3**) compared to oxalate-alone-treated cells (**Fig. 2G2**). A quantitative estimation of this decrease would lend more credibility to the observation. As a verification of the non-toxic effects of CP, treatment of the MDCK cells with this drug alone did not result in increased DCF fluorescence (**Fig. 2G4**) within the cells. Hence, we conducted a quantitative estimate of the DCF fluorescence and assessed the statistical significance (**Fig. 2B**), which revealed that the oxalate-treated cells exhibited an increased fluorescence (mean = 65) relative to control cells (mean = 12.4). The effects of CP treatment could be seen when the mean DCF fluorescence reduces to ∼28. Mean values of cells treated with CP alone were comparable to the controls (mean = 10.4). Taken together, our findings were suggestive of considerable attenuation of free radical generation in MDCK cells exposed to oxalate following pre-treatment with CP.

### C-phycocyanin Pre-Treatment Ameliorates Lipid Peroxidation in Oxalate-Treated MDCK Cells

**Figure 3:**
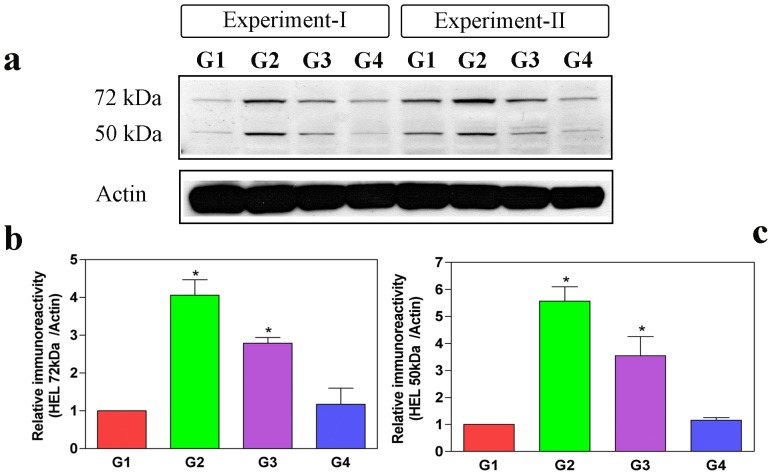
C-phycocyanin protects cells from free radical-mediated lipid peroxidation (LPO) induced by oxalate. To test the extent of free radical-mediated cellular damage, some oxidative stress markers have been developed and recognized suggestive of lipid peroxidation (LPO); for instance, HEL (hexanoyl-lysine adduct) is one the typical aldehyde products used as a marker of LPO. Thus, LPO was assessed by Western blotting for HEL in the lysates of MDCK cells from control (G1), oxalate alone (G2), oxalate+CP (G3) and CP alone (G4). Band intensity of HEL is prominent in oxalate-alone-treated cells (G2) at 72 kDa and 50 kDa compared to control MDCK cells (G1), whereas oxalate+CP cells (G3) showed decreased HEL band intensity relative to oxalate-treated cells (G2). The densitometric analyses were performed using a ImageJ (NIH) software. Densitometry data of 72 kDa and 50 kDa were normalized using internal control actin. Values represent mean±SD derived from immunoblots of all the groups from 3 experiments. The *P* values for HEL 72 kDa and 50 kDa of all the groups were as follows: HEL 72 kDa: G1 vs G2 (*P = 0.017) and G2 vs G3 (*P = 0.05); HEL 50 kDa: G1 vs G2 (*P = 0.013) and G2 vs G3 (*P = 0.009). P<0.05 were considered significant (*). G1: Control; G2: Oxalate; G3: Oxalate+CP; G4: CP alone.

Proteins modified with HEL are reportedly increased in proportion to the levels of LPO [34]. To investigate the level of LPO in oxalate-induced MDCK cells, we studied the Western blots of the total cell lysates of control (G1), oxalate-induced (G2), CP-treated plus oxalate induced MDCK cells (G3) and CP alone treated cells (G4) by using anti-HEL antibody (**Fig. 3a**). Predominant bands of ∼72 kDa and ∼50 kDa were noticeably increased in the oxalate-induced group as compared with the control group (**Fig. 3a–c**). However, the oxalate groups pre-treated with CP (G3) showed decreased levels of 72 kDa and 50 kDa protein bands compared to the oxalate-induced cell groups (G2) (**Fig. 3a–c**). Hence, we concluded that pre-treatment of CP alleviates LPO in oxalate-treated MDCK cells.

### Oxalate Disruption of Mitochondrial Trans-Membrane Potential [ΔΨ_m_] is Rectified by Pre-Administration of C-phycocyanin

**Figure 4:**
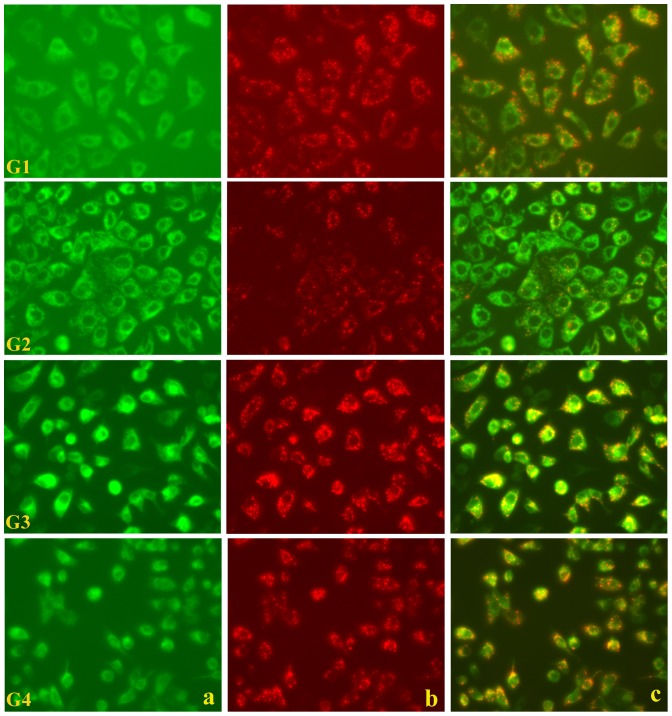
C-phycocyanin protects cellular mitochondrial membrane potential from oxalate-induced ROS. Integrity of mitochondrial membrane potential in MDCK cells investigated by a mitochondrial potential-sensitive dye, JC-1, showing normal stippled spots of bright red fluorescence (G1_a–c_). The numbers (viz. a–c) in the panels represents the red, green and merged channel fields observed respectively. Oxalate-exposed MDCK cells observed later (G2_a–c_) shows the absence of characteristic red fluorescence. Cells pre-treated with CP and oxalate showing the re-appearance of red fluorescence spots following normalization of mitochondrial potential (G3_a–c_). This confirms that CP could protect against mitochondrial membrane oxalate induced-ROS and does not itself affect the mitochondrial potential. Cells treated with CP alone show a normal picture (G4_a–c_). G1: Control; G2: Oxalate; G3: Oxalate+CP; G4: CP alone.

JC-1 is a ΔΨ_m_ sensitive fluorescent carbocyanine dye with a net positive surface charge [35], [36] that enables the dye to selectively accumulate in the mitochondria under conditions of intact trans-membrane potentials. This accumulation culminates in the aggregation of the dye within mitochondrial matrix that alters its fluorescent characteristics, which means that the monomeric form of JC-1 has a spectral emission maximum at 534 nm (green), shifted to 596 nm (red) on aggregation (dimer and multimeric forms). Thus a bright red fluorescence indicates an integral mitochondrial membrane with an intact trans-membrane potential (**Fig. 4G1_a–c_**). The fluorescence emanating from the mitochondria could be seen in red channel and when the red and green channels were merged, the intact and normal mitochondria stood out as stippled spots of bright red fluorescence. On the other hand, when the MDCK cell monolayer was incubated in a buffer containing oxalate and JC-1 and visualized, the red fluorescence was found to decrease (**Fig. 4G2_a–c_**). The merged fluorescence from both the red and green channels showed that there were hardly any intact mitochondria left as revealed by the marked absence of the bright red stippled spots seen earlier. The cellular architecture at this stage however, was not markedly disturbed, perhaps suggesting that the molecular events were at an initial stage. When the MDCK cells were pre-treated with CP and then incubated with oxalate and JC-1, and observed for mitochondrial membrane integrity, a remarkable restoration of mitochondrial trans-membrane potential was apparent (**Fig. 4G3_a–c_**). The effect of CP alone when applied to the MDCK cells was tested to rule out any deleterious effects of CP on the cells. The corresponding JC-1 fluorescence showed bright red stippled spots (**Fig. 4D_a–c_**) to clarify that CP might not have any cytotoxic effects *per se* and may not by itself compromise mitochondrial integrity at doses, which are known to attenuate oxalate toxicity.

### C-phycocyanin Pre-Treatment Increases ATP Levels in the Oxalate-Induced Cultured MDCK Cells

**Figure 5:**
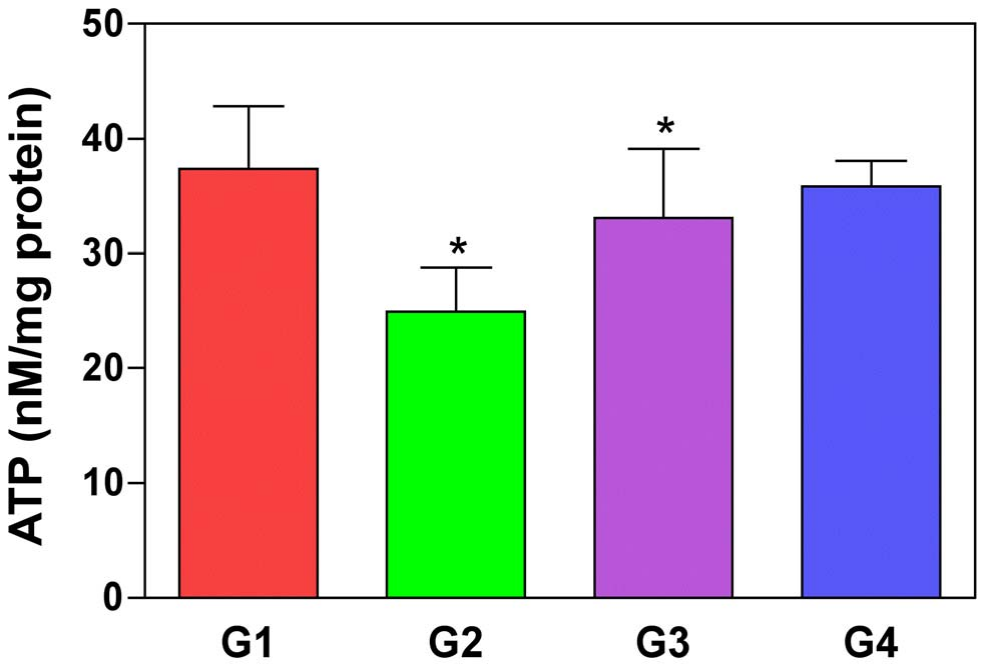
Oxalate-induced ATP depletion restored by C-phycocyanin pre-treatment in cells. ATP levels in cultured MDCK cells were significantly lowered when incubated with oxalate alone (G2) than control group (G1) (P = 0.02). ATP depletion was significantly restored when cells were pre-treated with CP under oxalate-induced conditions (G3) (P = 0.012). *G2 compared with G1; G3 compared with G2. P<0.05 were considered significant. G1: Control; G2: Oxalate; G3: Oxalate+CP; G4: CP alone.

A decrease in mitochondrial membrane potential is believed to be contributing to defective ATP synthesis [37], [38]. We tested the level of ATP production using a ATP bioluminescence assay [39] in oxalate-induced MDCK cells and compared it with control and CP treated cells (**Fig. 5**). We found that the ATP levels were significantly decreased in oxalate-induced MDCK cells (**Fig. 5G2**) (P = 0.002) compared to the control cells (**Fig. 5G1**). However, we also noticed that the decrease in ATP levels were significantly increased with combined oxalate and CP pre-treatment (**Fig. 5G3**) (P = 0.012). We also tested the effect of CP alone (**Fig. 5G4**) on cells to determine the possibility of toxicity of the compound, and found that the ATP levels were almost similar compared to control group ruling out the possibility of the effect of CP on ATP levels in normal cells. Hence, we concluded that pre-treatment of CP increases the levels of ATP in oxalate-induced culture of MDCK cells.

### C-phycocyanin Alleviates Oxalate-Induced Stress by Decreasing the Activation of ERK1/2 and JNK/SAPK in MDCK Cells

**Figure 6:**
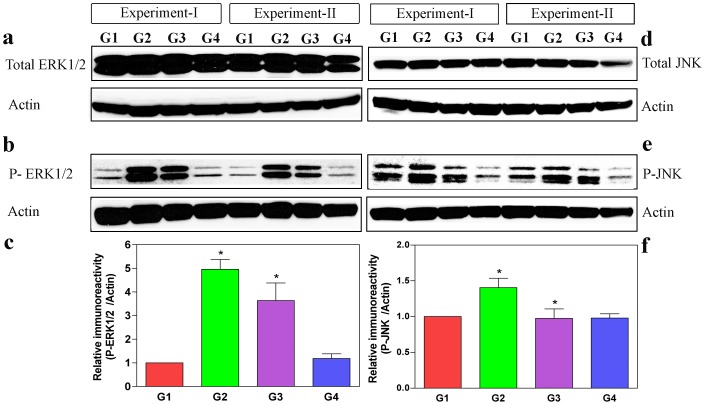
C-phycocyanin inhibits the activation of JNK and ERK1/2 in oxalate-induced cells. Activation of ERK1/2 and JNK in (MDCK cells) western blots of control (G1), oxalate-induced conditions (G2), oxalate+CP (G3) and CP alone (G4). The lysate RAB fractions were probed with antibodies for total and activated (phosphorylated) ERK1/2 and JNK. MDCK cells in all conditions had similar levels of total JNK and ERK1/2 (**a & d**). However, the levels of activated (phosphorylated) ERK1/2 and JNK were significantly lowered in oxalate+CP (G3) cell lysates than the oxalate alone (G2) groups (**b & e**). Scanning densitometry data of phosphorylated form of ERK1/2 and JNK were normalized using internal control actin. Phosphorylated ERK1/2 and JNK levels were significantly increased following oxalate-alone induction (G2), which was significantly reduced on pre-treatment with CP (G3) (**c & f**). However, the total ERK1/2 and JNK levels did not differ significantly in any of the groups investigated (Histogram data not shown). The Densitometric analyses were performed using a Image J NIH software. Values represent mean±SD derived from immunobots of all the groups from 4 experiments. The P values for activated (phosphorylated) ERK1/2 and JNK of all the groups were as follows: phosphorylated ERK1/2: G1 vs G2 (*P = 0.0025) and G2 vs G3 (*P = 0.027); phosphorylated JNK: G1 vs G2 (*P = 0.0089) and G2 vs G3 (*P = 0.015). P<0.05 were considered significant (*). G1: Control; G2: Oxalate; G3: Oxalate+CP; G4: CP alone.

Mitogen-activated protein kinases (MAPKs) play an important role in apoptosis signaling pathways [40]. Oxidative stress is known to activate members of the MAPK families (ERK and JNK) by protein phosphorylation [41]. To elucidate the link between damage due to oxalate-induced oxidative stress and subsequent activation of MAPKs, antibodies against JNK/SAPK were directed against the active phosphorylated forms of JNK, which recognizes all the three JNK isoforms, JNK1, JNK2 and JNK3. We investigated the effect of CP in oxalate induced MDCK cells on the phosphorylation of ERK1/2 and JNK by Western blot experiments. As shown in **Fig. 6a–f**, the levels of protein phosphorylation of ERK1/2 and JNK were increased in the oxalate-induced groups (G2), although the total JNK and ERK1/2 levels remained unaltered in all the experimental groups. The level of P-JNK/ and P-ERK1/2 levels were decreased in CP pre-treated oxalate-induced groups (G3) indicating the concrete role of CP in decreasing the oxalate-induced stress on MDCK cells.

## Discussion

The pathological hallmark of the present investigation is attributed to nephronal cell stress, which is initiated, maintained and facilitated entirely by free radical-induced chain of events [42]. Of note, this free radical assault by itself is believed to be elicited by oxalate [12]. Previous *in vivo* investigations on oxalate-treated Wistar rats have convincingly shown that their nephronal mitochondria contained high levels of the ROS [17]. Mitochondria being sites of aerobic respiration are also the sites of ROS synthesis, it is reasonable to hypothesize that it bears the maximum brunt of free radical-mediated cell injury. Determining if oxalate-mediated mitochondrial ROS generation could be targeted as a therapeutic strategy to ameliorate oxalate-induced nephrotoxicity in human kidney stone disease warrants a more detailed investigation, and therefore, herein we aimed to underpin the role of certain molecular events that are believed to be associated with free radical-induced mitochondrial injury.

### C-phycocyanin Alleviates Free Radical Burden of Mitochondria Following Oxalate Exposure Via its Antioxidant Effects

Free radicals such as H_2_O_2_ and superoxide anions are potent mediators of onset of LPO [43]. These effectors reportedly disconcert membrane integrity leading to the release of a plethora of mitochondrial activators of the cytosolic cysteinyl aspartate proteases (caspases) [44]. Thus it is important to recognize the role of cellular antioxidants like vitamin E in ameliorating cellular injury. Our previous studies have shown that CP helps in replenishing the levels of the antioxidants in cells subjected to oxalate exposure [22]–[24]. A constant pool of the antioxidants is thus maintained in the intracellular environment by CP [45]. When the MDCK cells were given oxalate and observed for the ROS levels, expectedly, there was a detectable rise in free radical activity. Our later observations in the present study pertaining to the effects of CP in protecting the ΔΨ_m_ could be the reason for the reduced ROS levels that were annulled by CP. In addition, perhaps the antioxidant effects of CP as mentioned above could reinforce its effects on membrane potential changes. Therefore, it can be conceptualized that CP has a credible role in restoring cellular antioxidant levels and protecting the cells from the stress induced by free radicals.

### C-phycocyanin Decreases Lipid Peroxidation Resulting From Oxalate Exposure

Oxalate induction is widely known to increase cellular LPO levels [46], [47]. Oxalate is a byproduct of LPO in cells, and therefore the levels of LPO could be correlated to the levels of HEL (hexanoyl-lysine adduct). Hence, in order to find out whether the increase of LPO induced following oxalate exposure could be reduced with CP treatment, we probed the Western blots of cell lysates from oxalate induced, control and CP-treated cells using antibodies against anti-HEL. Cells treated with oxalate showed distinctive bands at 72 kDa and 50 kDa indicating the increase in LPO. Groups treated with CP plus oxalate showed decreased 72 kDa and 50 kDa protein bands compared with oxalate induced groups, which confirms the notion that CP counteracts cellular metabolic processes like LPO induced by oxalate. Because, the production of HEL is implicated with increased oxidative stress in cells, the decreased levels of HEL with CP treatment provides a solid support to its role in ameliorating oxidative stress, which may be one of the mechanisms contributing to its therapeutic credentials in counteracting the effects of oxalate toxicity. These data further elucidate the role of CP in ameliorating oxidative stress and mitochondrial dysfunction caused by oxalate.

### C-phycocyanin Protects MDCK Cells from Oxalate-Induced Loss of Mitochondrial Membrane Potential (ΔΨ_m_)

In the present study we investigated whether the hitherto mentioned, and to some extent established, effects of oxalate on mitochondrial membrane permeability transition, could be corrected by CP treatment. The mechanisms by which oxalate is thought to detonate cell death pathways is still not clear, but it would be prudent to speculate that it proceeds through a preliminary extra-mitochondrial step involving certain cytosolic second messengers, and along with these mediators as well as independently, the mitochondrial membrane integrity may then be hit and the intra-mitochondrial apoptotic events involving ROS overproduction, follow. Therefore, it would be interesting to explore the specific steps of pathway initiation by oxalate in our future studies. The point to be noted here is that, oxalate could have direct roles in disrupting mitochondrial membrane integrity which could then trigger the ROS avalanche [48].

In our current study, we have observed the chain of events that occur on the introduction of oxalate into a culture of MDCK cells. The membrane permeability transition of the mitochondria seems to be an early indication of the onset of catastrophic events. Hence it makes more sensible to evaluate an ameliorating agent at this time-point. CP has been studied extensively for its antioxidant properties in a variety of clinicopathological settings and found to be a versatile pharmacological principle with a repertoire of beneficial effects including free radical scavenging and other therapeutic properties [49]–[53]. Hence we set out to see if this drug could restore the lost mitochondrial potential. The oxalate-induced changes of membrane potential have been found to occur approximately within 30 minutes of oxalate exposure of the cells [17]. Thus we surmised that this parameter would be reproducible and sensitive. Therefore we selected a dye JC-1 that was highly sensitive to any small changes in the ΔΨ_m_. This carbocyanine dye multimerizes within intact mitochondria and is easily detected by a shift in the emission spectrum to red from green. Our findings not only confirm that oxalate does indeed tamper with the mitochondrial membrane integrity, but also show that CP can negate these effects. It could be interesting to investigate the steps in the cytoplasm, which might precede the eventual onset of mitochondrial events including changes in the trans-membrane potential.

The exposure of cells to oxalate induced a transition in the mitochondrial membrane permeability as we observed from an earlier experiment using the JC-1 dye. In healthy cells, the dye stains the mitochondria bright red. The negative charge established by the intact mitochondrial membrane potential allows the lipophilic dye, bearing a delocalized positive charge, to enter the mitochondrial matrix where it accumulates. When the critical concentration is exceeded, JC-1 aggregates form that become fluorescent red (absorption/emission maxima of 585/590 nm). In apoptotic cells, the mitochondrial membrane potential collapses forbidding JC-1 to accumulate within the mitochondria. In these cells JC-1 remains in the cytoplasm in a green fluorescent monomeric form (absorption/emission maxima of 510/527 nm). Apoptotic cells, showing primarily green fluorescence, are easily differentiated from healthy cells, which show red and green fluorescence. Oxalate treatment was found to alter this ratio of green to red fluorescence and this indicates that there was a measurable decrease indeed in the trans-membrane potential of the mitochondria upon exposure to oxalate. It is also to be noted that in line with our DCFDA observations pertaining to the effects of CP on the oxalate treated cells, here also it could be appreciated that there is a measurable restoration of the mitochondrial potential due to CP, lending credence to our earlier observations.

### Restoration of Oxalate-Induced Decrease in ATP Levels with C-phycocyanin in MDCK Cells

**Δ**Ψ_m_ is the driving force behind ATP production [54]. Mitochondria generate ATP by utilizing the proton electrochemical gradient potential generated by serial reduction of electrons through the respiratory electron transport chain (ETC). So a decrease in the mitochondrial membrane potential is associated with defects in aerobic respiration and reduced ATP synthesis [55]. So we hypothesized that oxalate may cause a decrease in ATP levels as it affects the mitochondrial membrane permeability. We quantified ATP levels of oxalate induced MDCK cells with ATP bioluminescence assay and compared it with control, oxalate & CP-induced combination groups. Our data indicated that oxalate induces a decrease in the ATP levels of the cells which are restored by the CP treatment. This shows that oxalate induced ATP reduction in the cells can be counteracted by CP inhibiting the effects of oxalate on cellular metabolic processes in the cells.

### Activation of Stress-Induced MAP Kinases May Explain Oxidative Stress and Mitochondrial Damage in Oxalate-Induced MDCK Cells

The JNK and ERK1/2 pathways play a critical role in response to cellular stress by promoting cell growth and survival [56]. Therefore, we investigated the effect of oxalate on MAPK signaling pathways by directing antibodies against phosphorylated forms of JNK/SAPK and ERK1/2. Our results demonstrate that oxalate stimulates JNK and ERK1/2. Activation of the stress induced kinase pathway results in a plethora of changes in transcription, protein synthesis, cell surface receptor expression, and cytoskeletal structure, ultimately affecting cell survival leading to programmed cell death [57]. Thus activation of the MAPK cascade is suggestive of a functional role of this kinase cascade in mediating cellular actions of oxalate. Cellular stress activates major stress-activated signaling pathway with regard to p38 MAPK, Serine-threonine protein kinases, as well as the JNK family [58]. JNK is activated by osmotic stress and during ischemia-reperfusion of the kidney [59]. Reports suggest the involvement of JNK in apoptotic signals [57]. It has also been shown that extracellular stress-related kinase activation inhibits apoptosis, whereas cytokine induced apoptosis is mediated by JNK [60]. In our in vitro study, the disappearance of JC-1 staining indicated mitochondrial collapse through depolarization of the mitochondrial membrane. We hypothesize that oxalate induced mitochondrial injury preceded a change in gene expression in the renal tubular cells. Decreased ATP levels, increased ROS and HEL levels indicated an increase in oxidative stress in renal tubular cells. Increased stress induced kinases JNK and ERK1/2 expression, decreased ATP levels and decreased cell viability were indicative of mitochondrial collapse and loss of mitochondrial membrane potential through induction of the apoptotic pathway by oxalate injury.

In a nutshell, one can therefore construe about essentially two broad concepts, namely oxalate-induced oxidative stress and CP induced anti-oxidative response, which can be a part of the overarching hypothesis that tries to explain the pathogenic mechanisms of oxalate-induced nephrotoxicity and the role of antioxidant defense measures in countering the detrimental consequences of oxalate. Our present study is one of the first steps to show that oxalate induces increased ROS production, which might themselves mediate mitochondrial membrane permeability transition culminating in a leaky mitochondrion. Our study throws light on the sites where CP might be active in that, the endogenous pool of cellular antioxidants goes up and at a molecular level, the levels of mitochondrial membrane potential and ATP production are enhanced and cell stress is aborted by CP. Further studies to strengthen our current observations that CP could significantly prevent oxalate-mediated LPO and cellular stress-induced MAP kinases may be warranted. Therefore, our current investigation marks the beginning of an exciting novel therapeutic strategy against oxidative stress-associated diseases.

## Materials and Methods

### Chemicals and Reagents

Oxalate, C-phycocyanin (CP), DMEM (Dulbecco's Modified Eagle Medium), fetal bovine serum (FBS), propidium iodide (PI), trypan blue, MTT (3-(4,5-dimethylthiazol-2-yl)-2,5-diphenyltetrazolium bromide), dimethyl sulfoxide, trypsin, Tween 20, and Triton X-100 were purchased from Sigma Chemicals (St. Louis, MO, USA). 2, 7–dichlorofluorescein diacetate (DCF-DA) (Molecular probes, Inc. USA), and JC-1 dye (Molecular probes, Inc. USA) were procured from Molecular Probes, Inc. (USA), P-JNK/SAPK and P- ERK1/2 antibodies from Cell signaling Technology, USA. T-JNK and T-ERK1/2 antibodies were purchased from Santa Cruz Biotechnology, INC, USA.

### MDCK Cells

Renal epithelial cells of the MDCK cell line was maintained in complete DMEM medium supplemented with 10% FBS and penicillin-streptomycin. The cells were maintained at 37°C in a humidified atmosphere of 95% air and 5% CO_2_. Different concentrations of CP were prepared including 5, 10, 15, 20, 50 mM from which an effective dosage was optimized for consideration in the treatment of oxalate toxicity. Oxalate concentration in the medium was fixed according to Scheid et al. (1996) [61] at 0.02, 0.04, 0.06, 0.08 and 0.1 mM; effective dosage was used to induce oxalate toxicity. The cells (1×10^5^) were incubated with the medium containing the aforementioned concentrations of oxalate and CP.

### Cell Viability

Cell viability was determined by quantitative colorimetric MTT assay [62]. The assay was based on the ability of mitochondria from viable cells to cleave the tetrazolium rings of the pale yellow MTT resulting in the formation of a dark blue formazan product. The cells were treated in triplicates with oxalate and with or without CP (treated 1 hour prior to oxalate induction) (5, 10, 20, and 50 mM) for 24 hours. At the end of each time point, 20 µL of MTT (5 mg/mL) was added to each well and the plates were incubated for 4 hours at 37°C. The MTT formazan precipitate was subsequently dissolved in dimethyl sulfoxide (DMSO) and read under a microplate reader (Dynatech, Chantilly, Va., USA) at a test wavelength of 562 nm and a reference wavelength of 660 nm.

### Determination of ROS Levels

Intracellular ROS production was monitored using 2,7–dichlorofluorescein diacetate (DCFDA; Molecular probes, Inc. USA, Cat. No. C6827). DCFDA is hydrolyzed by non-specific cellular esterases and subsequently oxidized by ROS to form a fluorescent product, 2′7′-dichlorofluorescein (DCF). MDCK cells were incubated with 5 µM DCFDA for 20 min and washed three times with PBS. Levels of intracellular ROS were subsequently determined by image analysis of DCFDA loaded cells on a fluorescent microscope (Olympus IX70, Japan). DCFDA is membrane permeable and interacts with ROS (emission green) in living cells.

### Western Blot Analysis of Hexanoyl-Lysine Adduct

Forty micrograms of MDCK cell lysates were resolved by sodium dodecyl sulfate-polyacrylamide gel electrophoresis (SDS–PAGE) using 5–20% gel [63], [64]. Following electrophoretic resolution, the proteins were transferred onto a nitrocellulose membrane using an electrophoretic system (BioRad, Germany). The membrane was incubated in 5% skimmed milk at room temperature for 1 hour, rinsed, and reacted with primary antibody (1∶250 dilutions) against 4-HEL (Cat. No. MHL-020P; Clone 5H4; Japan Institute for the Control of Aging, JaICA, Japan) in Tris buffered saline (TBS) overnight. The membrane was washed six times in TBST and incubated with horseradish peroxidase (HRP)-labeled secondary antibody (1∶2000) for 1 hour. After six washes with TBST, the immunoreactive bands were visualized using the ECL developing kit [65].

### Determination of Mitochondrial Membrane Potential (JC-1 Staining) by Fluorescence Microscopy

MDCK cells were incubated in a medium containing 0.5 µM JC1 dye (Molecular probes, Inc. USA, Cat. No. T-3168) for 30 minutes at 37°C and washed with PBS. Subsequently, the cells were excited at 488 nm and the fluorescence emission was recorded at 530 nm and 560 nm. Images were captured using a CCD camera (Hamamatsu Digital Camera; Meta Vue software) and 1280×1024 pixel images were collected. The levels of green and red fluorescence were coded on scale (0–4095) representing pixel intensity. The stock solution of JC1 was dissolved in 100% DMSO, and the final concentration of DMSO used was <0.1% [66].

### Determination of Cellular ATP

ATP levels were determined using a commercial ATP Bioluminescence Assay kit CLS II (Boehringer Mannheim, Mannheim, Germany). Briefly, the MDCK cells were sonicated in PBS, pelleted by centrifuging at 10000 rpm (9200 g) for 2 minutes at 4°C, washed with PBS, resuspended in 50 µL of ice-cold ATP lysis buffer (100 mM Tris and 4 mM EDTA, pH 7.75) and incubated for 2 minutes at 99°C in 150 µL of boiling ATP lysis buffer. Cell lysates were centrifuged at 10000 rpm for 1 minute at 4°C. ATP was measured using 20 µL of the supernatant and 80 µL of luciferase reagent. After a 20 second delay, the chemiluminescence was measured with 2 seconds integration time using ARVO MX/Light (1420 Multilabel Counter, Perkin Elmer).

### Immunoblotting for Kinases

Method for RAB fraction of cell lysate was described previously [66]. Briefly, the MDCK cells were homogenized in 4 mL/g of RAB buffer (0.1 M MES, 1 mM EGTA, 0.5 mM MgSO4, 150 mM NaCl, 0.02M NaF, 1 mM PMSF, phosphatase and protease inhibitor, pH 7.0) and centrifuged at 40,000 g for 20 minutes at 4°C (RAB fraction). The RAB fractions were probed differentially with the following antibodies against P-JNK/SAPK (Cat. No. 9251), P- ERK1/2 (Cat. No. 9101) (Cell signaling Technology, USA), and T-ERK1 (K-23; sc-94) and T-JNK (F-3; sc-1648) (Santa Cruz Biotechnology, INC, USA).

### Statistical Analysis

Data were expressed as mean ± SD. The student *t* test was used to evaluate the significance of these experiments. Statistical analysis was performed with GraphPad Prism 5 software (GraphPad Software, Inc., La Jolla, CA, USA).
