# Correction: C-Phycocyanin Confers Protection against Oxalate-Mediated Oxidative Stress and Mitochondrial Dysfunctions in MDCK Cells

**DOI:** 10.1371/journal.pone.0103361

**Published:** 2014-07-17

**Authors:** The PLOS ONE Staff

The third author's name is spelled incorrectly. The correct name is: Asokan Devarajan. The correct citation is: Farooq SM, Boppana NB, Devarajan A, Sekaran SD, Shankar EM, et al. (2014) C-Phycocyanin Confers Protection against Oxalate-Mediated Oxidative Stress and Mitochondrial Dysfunctions in MDCK Cells. PLoS ONE 9(4): e93056. doi:10.1371/journal.pone.0093056
